# Borax and Boracic Acid in Otorrhœa

**Published:** 1882-05

**Authors:** 


### BORAX AND BORACIC ACID IN OTORRHŒA.

In the treatment of chronic inflammation of the middle ear with destruction of the drum-head, Professor Gruber, of Vienna, uses a concentrated solution of borax. When the inflammation is suppurative in character, filling the canal with powdered boracic acid gives very good results. To prevent relapses, the treatment must be continued for several weeks after all discharge has ceased. The boracic acid should be finely powdered.

